# Analysis of relative factors and prediction model for optimal ovarian response with gonadotropin-releasing hormone antagonist protocol

**DOI:** 10.3389/fendo.2022.1030201

**Published:** 2022-11-15

**Authors:** Wenwen Jiang, Beihong Zheng, Xiuhua Liao, Xiaojing Chen, Suqin Zhu, Rongshan Li, Huale Zhang

**Affiliations:** ^1^ Center for Reproductive Medicine, Fujian Maternity and Child Health Hospital, Affiliated Hospital of Fujian Medical University, Fuzhou, China; ^2^ Obstetrics and Gynecology Department, Fujian Maternity and Child Health Hospital, Affiliated Hospital of Fujian Medical University, Fuzhou, China

**Keywords:** GnRH antagonist protocol, controlled ovarian hyperstimulation, ovarian response, nomogram prediction model, obtained eggs

## Abstract

**Objective:**

To explore the relative factors for best ovarian response in patients undergoing assisted reproductive technology with the gonadotropin-releasing hormone antagonist protocol and to establish a nomogram prediction model of ovarian response.

**Methods:**

A retrospective cohort analysis of the clinical data of 1,944 patients who received assisted reproductive treatment in the Center for Reproductive Medicine of Fujian Maternity and Child Health Hospital from April 1, 2018, to June 30, 2020. According to the number of oocytes obtained, there were 659 cases in the low ovarian response group (no more than five oocytes were retrieved), 920 cases in the normal ovarian response group (the number of retrieved oocytes was >5 but ≤18), and 365 cases in the high ovarian response group (>18 oocytes retrieved). Independent factors affecting ovarian responsiveness were screened by logistic regression, which were the model entry variables, and a nomogram prediction model was established based on the regression coefficients.

**Results:**

There were statistically significant differences in age, anti-Mullerian hormone, antral follicle count, the diagnosis of endometriosis, decreased ovarian reserve, polycystic ovary syndrome, basal follicle-stimulating hormone and basal luteinizing hormone among the three groups (P < 0.001). Multifactorial stepwise regression analysis showed that female age (0.95 [0.92–0.97], *P* = 0.000), decreased ovarian reserve (0.27 [0.19-0.38]), *P* = 0.000), endometriosis (0.81 [0.56-0.86], *P* = 0.000), antral follicle count (1.09 [1.06-1.12], *P* = 0.000), basal follicle-stimulating hormone (0.90 [0.85-0.96], *P* = 0.001), Anti-Mullerian hormone (1.19 [1.13–1.26], *P*= 0.000) and luteinizing hormone on trigger day (0.73 [0.66–0.80], *P*= 0.000), were independent factors for the occurrence of different ovarian responses during ovarian hyperstimulation. The predictive model of ovarian responsiveness was constructed based on the above factors, and the model was verified with 589 patients’ data from July 1, 2020, to December 31, 2020, at this center. The predicted ovarian response (number of eggs obtained) of a total of 450 patients was consistent with the actual results, with a coincidence degree of 76.4%, and the consistency index of the model is 0.77.

**Conclusion:**

The nomogram model was successfully developed to effectively, intuitively, and visually predict the ovary reactivity in the gonadotropin-releasing hormone antagonist protocol and provide guidance for clinical practice.

## Introduction

Controlled ovarian hyperstimulation (COH) is an important step in assisted reproductive technology. Obtaining the appropriate number of eggs of good quality after fertilization, to form high-quality embryos implanted into the uterine cavity, is key in pregnancy (1). Ovarian reactivity is the sensitivity of the ovary to exogenous gonadotropin (Gn) during COH. The reactivity of the ovary determines whether the appropriate number of oocytes can be recruited, which is one of the factors for success of COH (2), and directly affects the whole ovulation induction process and the outcome of assisted reproduction. Ovarian reactivity can be divided into three categories: low ovarian response; normal ovarian response; and high ovarian response. The ovaries respond poorly to Gn stimulation, with a small number of eggs harvested, called a low response. On the contrary, the ovary is extremely sensitive to Gn stimulation, which exceeds the expected level and obtains too many eggs, which is called ovarian hyper-response, and is the most important factor that may cause ovarian hyperstimulation syndrome.

Gonadotropin-releasing hormone (GnRH) antagonist has become increasingly popular in clinical practice due to its advantages of convenient use, flexibility, and fewer side effects, and has become a mainstream classical clinical program (3, 4). This protocol removed the down-regulating effect of a long recovery period and effectively reduced the occurrence of ovarian hyperstimulation syndrome, which greatly improved the safety of *in vitro* fertilization and embryo transfer (IVF-ET) treatment. At the same time, the treatment period of GnRH antagonist is shorter, the dosage of Gn is lower, the ovarian function recovers quickly, and patient satisfaction is higher than with GnRH agonist (5, 6). However, because of the shallow inhibition of the pituitary gland, early luteinizing hormone (LH) peak may occur and lead to early ovulation (7). Therefore, it remains necessary to prescribe a suitable starting and total dose of Gn to obtain good ovarian response and avoid ovarian overstimulation in the process of COH. Too low a starting dose may artificially induce a low response in the ovaries and, conversely, there is a risk of a high response (8). Of course, it is also important to adjust the dose of Gn according to the ovarian response during ovulation and to add antagonists when appropriate.

Anti-Mullerian hormone (AMH), inhibin B, age, antral follicle count (AFC), basal sex hormones, etc. are commonly used to predict ovarian responsiveness in clinical practice (9, 10). These indicators have limitations in predicting ovarian responsiveness, the cut-off values are not standardized (11), and it is not possible to predict ovarian responsiveness as a whole by individual indicators in a single patient. We aimed to screen for independent risk factors affecting ovarian responsiveness in GnRH antagonist by stepwise regression and to establish a nomogram model to predict ovarian responsiveness based on the regression coefficients of these variables. Each woman undergoing *in vitro* fertilization/intracytoplasmic single sperm microinjection-embryo transfer (IVF/ICSI-ET) was offered an individualized ovulation process to achieve the right number of oocytes and improved pregnancy outcomes.

## Materials and methods

### Patient population and study design

This study was approved by the Ethics Review Committee of Fujian Provincial Maternal and Child Health Hospital (Approval No. 2021YJ037). Female patients attending the Fertility Center of Fujian Provincial Maternal and Child Health Hospital for fertility treatment between April 1, 2018, and June 30, 2020, were selected for the retrospective cohort study. Inclusion criteria were: age, 20–40 years; the patients were in the GnRH antagonist protocol and infertility due to tubal factors, polycystic ovary syndrome(PCOS), endometriosis (EMT), decreased ovarian reserve(DOR), male factors and other factors;. Exclusion criteria were: patients with other protocols; the ovulation induction cycle was cancelled and comorbidity with other systemic diseases such as Cushing’s syndrome, pituitary tumors, and other systemic diseases; and pre-implantation genetic testing.

Based on a literature search and the different transfer strategies adopted by our center, we defined ovarian responsiveness based on the number of eggs obtained. The low response group was defined as ≤5 eggs obtained; normal response group, >5 and ≤18; and high response group, >18 (12, 13).

### Ovarian stimulation

The patients adhered to the GnRH antagonist protocol. On the second to third day of the menstrual cycle, the size and number of bilateral antral follicles were monitored by B ultrasound, and the starting dose of recombinant follicle-stimulating hormone (rFSH, Gonal-F, Merck Serono, Switzerland) was determined by the physician based on the patient’s basal endocrine level, AFC,AMH, age, and body mass index (BMI), to start ovulation promotion, usually at a dose of 150–300 IU/d. Ovulation was monitored by vaginal ultrasound on day five of rFSH injection, and antagonists were added according to follicle size and serum estrogen and LH levels. We used a flexible protocol for the addition of antagonists (14). Patients were given subcutaneous cetrorelix acetate (Stryker, Merck Serono, Switzerland), 0.25 mg daily until human chorionic gonadotropin (HCG) day. When at least two follicles were ≥18 mm in diameter, or more than 50% of follicles were ≥16 mm in diameter, 250 ug of recombinant HCG (r-HCG, Azer, Merck Serono, Switzerland) or HCG (Maanshan Fengyuan Pharmaceutical Co., Ltd, Anhui, China) 6000–10,000 U were injected as a trigger. The decision of which trigger drug to use was based on the patient’s E2 level and the number of dominant follicles on triggering day. The eggs were retrieved under vaginal ultrasound guidance, 36–38 h after HCG injection. Sperm retrieval was done from the male partner on the day of egg retrieval for IVF or ICSI (only in cases of severe male factor infertility). Progesterone injection (Zhejiang Xianju Pharmaceutical Co., Ltd. Zhejiang, China), 40 mg/d and didrogestrel tablets (Daphne 10 mg/tablet, Solvay Pharmaceuticals, Netherlands), 10 mg, bid, were prescribed for luteal support after egg retrieval.

The oocytes were cultured *in vitro* for 3–6 h, subjected to conventional IVF or ICSI, and the best quality embryos were selected for transfer under abdominal ultrasound guidance on the third day after fertilization. No more than two embryos were transferred at a time. The remaining oocytes were cultured for blastocysts, which were frozen. There were no cases of fresh blastocyst transfer in this study. If more than 18 eggs were obtained, the patient required vaginal ultrasound on the day of transplantation. If the number of eggs obtained exceeded 20, fresh embryo transfer was not possible, and only considered in special cases.

Post-transplantation luteal support was performed with progesterone vaginal extended-release gel (Certolone 90 mg/d, Merck Serono, Switzerland), 8% per day and didrogesterone tablets, 10 mg, bid. Blood HCG was checked 14 days after transplantation and, if >5 mIU/ml, biochemical pregnancy was considered. An ultrasound examination was performed approximately one month after transplantation, and clinical pregnancy was diagnosed when germ and heart tube pulsation were seen. Luteal support was maintained until 10–11 weeks of gestation.

### Statistical analysis

SPSS 22.0 and R3.5.1 software were used to analyze the data. The measurement data were expressed as mean ± standard deviation (x ± s), and a t-test or Kruskal–Wallis test was used to compare the data between groups. Count data are expressed as rate (%), and the χ2 test or Fisher’s exact test was used for comparison between groups. Statistically significant baseline information from each data was included in a stepwise regression analysis to screen for independent risk factors affecting ovarian responsiveness. R3.5.1 software was used to establish the Norman model (15). The performance of the prediction model was constructed by Harrell’s concordance (consistency index-c index). Statistical significance was accepted when P < 0.05.

## Results

### Baseline characteristics

A total of 1,944 patients were enrolled, including 659 cases in the low response group, 920 cases in the normal response group, and 365 cases in the high response group. Baseline characteristics of the patients are shown in **Table 1**. There were significant differences in age, AMH value, and AFC among the three groups (all P < 0.001), but there were no significant differences in BMI and infertility years among the three groups (all P > 0.05).

**Table 1:** Comparison of baseline data of patients with different ovarian reactivity.

Item	Low ovarian response group	Normal ovarian response group	High ovarian response group	Z/χ^2^ value	*P* value
No. of cases	659	920	365	/	/
Age (year)	36 (32,40)	31 (29,35)	29 (27,31)	372.164	<0.001
BMI (kg/m^2^)	21.64 (19.97,23.76)	21.63 (19.94,23.73)	21.10 (19.57,23.44)	6.752	0.054
Duration of infertility (year)	3 (2,5)	3 (2,5)	3 (2,5)	5.168	0.075
Infertility reason (%)					
DOR	71.47 (471/659)	16.74 (154/920)	0.00 (0/365)	740.228	<0.001
PCOS	1.52 (10/659)	12.39 (114/920)	35.89 (131/365)	244.328	<0.001
EMT	21.24 (140/659)	12.17 (112/920)	4.11 (15/365)	61.798	<0.001
AMH (μg/L)	0.98 (0.65,1.56)	3.42 (1.91,5.88)	7.94 (5.87,11.75)	1029.556	<0.001
AFC	6 (4,8)	13 (9,19)	23 (19,28)	962.525	<0.001
Basal FSH (IU/L)	7.54 (6.15,9.5)	6.03 (5.13,7.19)	5.14 (4.44,6.02)	393.734	<0.001
Basal LH (IU/L)	2.9 (2.1,3.9)	3.4 (2.5,4.7)	4.4 (3.2,6.2)	177.106	<0.001
Gn initial dose (IU)	225 (225,225)	225 (150,225)	150 (125,187.5)	601.711	<0.001
Total dosage of Gn used (IU)	2250 (1875,2700)	2100 (1725,2550)	1650 (1362.5,2175)	185.424	<0.001
Duration of Gn used (d)	9 (8,11)	10 (9,11)	11 (10,12)	132.727	<0.001
Estradiol level on hCG injection day (ng/L)	879 (586.5,1383)	2807 (1894.75, 3939.25)	5708 (4414, 7807)	1197.769	<0.001
No. of oocytes retrieved	3 (2,4)	10 (8,13)	23 (20,28)	1652.807	<0.001

BMI represents Body mass index, DOR represents Decreased ovarian reserve, PCOS represents Polycystic ovary syndrome, EMT represents Endometriosis, AMH represents Anti-Mullerian Hormone, AFC represents Antral follicle counting, FSH represents Follicle-stimulating hormone, LH represents Luteinizing hormone, Gn represents Gonadotropin, HCG represents Human chorionic gonadotropin, positive number/total number in brackets.

### Data related to COH

The ovulation induction regimen of the patients included in this study was that of the GnRH antagonist protocol. As can be seen from **Table 1**, the starting doses of COH for the three groups of patients with different reactions were 225.0 IU, 225.0 IU, and 150.0 IU, respectively, and the differences were statistically significant (P < 0.001). The time of Gn used was 9 days, 10 and 11 days, respectively, and the difference was also statistically significant (P < 0.001). Therefore, the difference in total dose of Gn use was statistically significant (P < 0.001). Similarly, during ovulation induction, estradiol levels on HCG days were highest in the high ovarian response group and lowest in the low ovarian response group. The number of eggs retrieved was significantly different among the three groups (P < 0.001) (**Table 1**). This can be regarded as a key finding from our cohort.

### Pregnancy outcome analysis

Excluding the patients who did not undergo transfer for various reasons, the clinical pregnancy rates of the three groups were 35.8%, 41.2%, and 42.6%, respectively, with no statistically significant difference (P = 0.287). The rate of ectopic pregnancy in the normal response group was 1.5%, and there were no ectopic pregnancies in the other two groups. The live birth rates were 28.1%, 34.5%, and 27.7%, with no statistically significant difference (P = 0.158). Among these patients, seven cases of mild ovarian hyperstimulation syndrome (OHSS), 20 cases of moderate OHSS, and 21 cases of severe OHSS occurred, and the total incidence of OHSS was 2.47%. Among moderate OHSS, one case was in the low response group, 4 in the normal response group, and 15 in the high response group. In the severe OHSS group, there were 7 cases in the normal response group and 14 cases in the high response group. In the low ovarian response, there were no incidences of OHSS (**Table 2**).

**Table 2:** Indicators related to COH outcomes in patients with different ovarian reactivity.

Item	Low ovarian response group	Normal ovarian response group	High ovarian response group	Z/χ^2^ value	*P* value
No. of cases	659	920	365	/	/
Clinical pregnancy rate (%)	35.8 (112/313)	41.2 (171/415)	42.6 (23/54)	2.494	0.287
Ectopic pregnancy rate (%)	0 (0/313)	1.5 (6/415)	0 (0/54)	/	/
Live birth rate (%)	28.1 (88/313)	34.5 (143/415)	27.7 (15/54)	3.694	0.158
Percentage of moderateOHSS (%)	0.2 (1/659)	0.4 (4/920)	4.1 (15/365)	/	/
Percentage of severe OHSS (%)	0 (0/659)	0.8 (7/920)	3.8 (14/365)	/	/

COH represents Controlled ovarian hyperstimulation; OHSS represents Ovarian hyperstimulation syndrome; positive number/total number in brackets.

### Modeling group variables

Multiple variables included in the model were screened by stepwise regression. The results showed that female age, decreased ovarian reserve, EMT, basal AFC, basal follicle-stimulating hormone (FSH) value, AMH value, and LH value on HCG day were independent factors for ovarian normal response (**Table 3**).

**Table 3:** Parameter variables screened by multi-factor stepwise regression.

Variable value	*P* value	*OR*	95% *CI*
Female’s age	0.000	0.95	0.92~0.97
DOR	0.000	0.27	0.19-0.38
EMT	0.000	0.81	0.56-0.86
AFC	0.000	1.09	1.06~1.12
Basal FSH	0.001	0.90	0.85~0.96
AMH	0.000	1.19	1.13-1.26
LH value on HCG day	0.000	0.73	0.66~0.80

DOR represents Decreased ovarian reserve, EMT represents Endometriosis, AFC represents Antral follicle counting, AMH represents Anti-Müllerian hormone, FSH represents Follicle-stimulating hormone, LH represents Luteinizing hormone.

### Establishment of the Norman model

**Figure 1:**
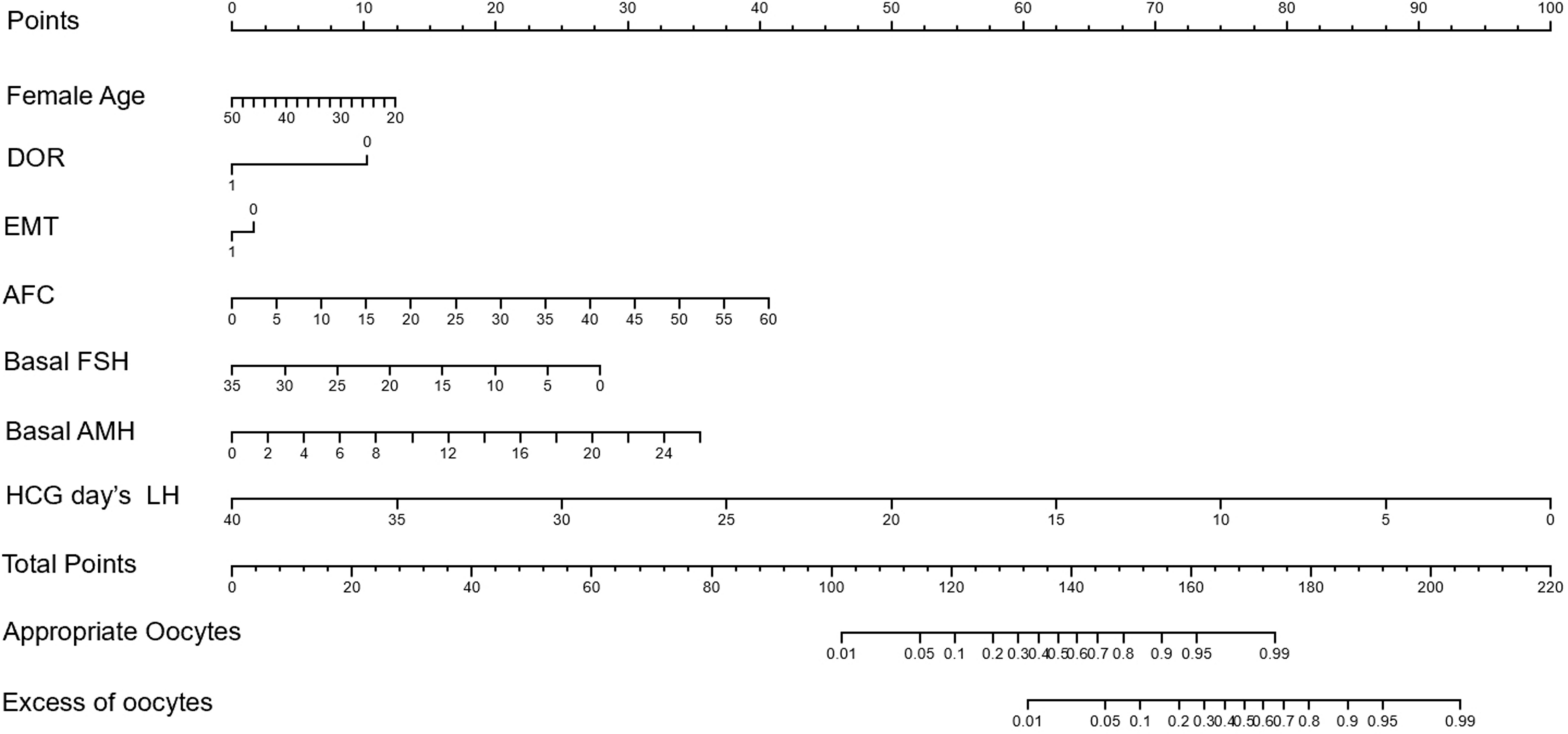
Nomogram model for the prediction of optimal ovarian response and hyperresponsiveness in patients treated with GnRH antagonist protocol.

The Norman model was successfully established according to the results of the stepwise regression analysis. The values on the scale line of each predictor corresponded to the score on the scale line, and the scores of all indicators were summed to obtain the total score. The total score corresponded to the predicted risk value (the probability of having an appropriate number of follicles or an excess number of follicles). Finally, the nomogram model for predicting ovarian response was established, as shown in **Figure 1**.

### Validation of the Norman model

The data of 589 similar patients who visited our center from July 1, 2020, to December 31, 2020, were selected to verify the above model. After the seven variables were included, the probability of obtaining an appropriate number of follicles and the probability of obtaining excessive number of follicles were calculated. The predicted oocyte retrieval was compared with the actual oocyte retrieval to verify the ability of the model to predict the best ovarian response. The results showed that 164 of the 204 patients with low response were accurately predicted. Of the 277 best response patients, 216 were accurately predicted. The prediction was accurate in 70 of the 108 patients who actually had a high response. In other words, the predicted ovarian response (number of eggs retrieved) was consistent with the actual outcome in 450 of 589 patients, with an accuracy of 76.4%. The concordance index (C-index) of this model was 0.77, which was regarded as good accuracy.

## Discussion

There were statistically significant differences in age, AMH, AFC, diagnosis of EMT, DOR, PCOS, basal FSH, and basal LH among the three groups. Multifactorial stepwise aggression analysis showed that female age, DOR, EMT, AFC, basal FSH, LH on trigger day, and AMH were independent factors for the occurrence of different ovarian responses during ovarian hyperstimulation. The prediction model of ovarian responsiveness was constructed based on the above factors, the model was verified with patient data, and the predicted ovarian response was consistent with the actual results, with a coincidence degree of 76.4%.

### GnRH antagonist protocol and ovarian responsiveness

GnRH antagonists block the production of endogenous Gn and rapidly reduce the levels of endogenous luteinizing and follicle-stimulating hormone by competitively binding to the receptor of endogenous GnRH, thus effectively inhibiting the occurrence of endogenous LH peak. This means that the pituitary gland can still stimulate the development and maturation of multiple follicles without being down regulated. This competitive binding is reversible, and inhibition of the pituitary gland can be relieved 48 hours after removal of the drug. The GnRH antagonist protocol has been widely used because of its simplicity of operation and reduced number of visits to the hospital for assisted reproductive patients. In the first three days of menstruation, with appropriate antral follicle size and sex hormones, ovulation induction was performed using the appropriate Gn initiation dose and optimizing the total Gn dose. This can shorten the time to reach pregnancy to the greatest extent, and improve the efficiency and economic benefits to patients. In recent years, our center has expanded the range of utility of the GnRH antagonist protocol; it is not limited to patients with DOR or polycystic ovary syndrome, but is also used for patients with tubal infertility, male factors, advanced age, and other infertility factors, and fresh cycle transfer should be performed as far as possible. The results of this paper show that the oocyte retrieval rate and clinical pregnancy rate of this protocol remain relatively stable.

In the prevention of OHSS, the antagonist protocol is more effective than the long-acting protocol in the follicular phase (16). It is well known that OHSS is a common and serious iatrogenic complication after ovulation induction (17). During assisted reproduction, approximately 20–30% of patients will have different degrees of OHSS symptoms (18). The incidence of moderate OHSS is approximately 3–6%, while the incidence of severe OHSS is about 0.1–2% (19). The results of this study showed that the overall incidence of moderate OHSS was 1.02%, which was much lower than that reported in previous studies, while the incidence of severe OHSS was 1.08%, which was at a low level compared to that reported in other studies. However, in the high response group, the incidence of moderate OHSS (4.1%) and severe OHSS (3.8%) was much higher than that in the normal and the low response groups, which was also significantly related to the oocyte retrieval rate. Therefore, we hope that during ovulation induction, the ovaries will be at the optimal response level to obtain the appropriate number of follicles and reduce the incidence of complications.

### Factors associated with ovarian responsiveness

Traditionally, age, AFC, inhibin B, FSH and AMH have been used to predict ovarian responsiveness. Age is known to be an important predictor of ovarian responsiveness and egg quality, especially in elderly women. However, there are some limitations, perhaps because there are individual differences in ovarian responsiveness which cannot be determined by age alone. The lower value of age in the prediction of ovarian responsiveness in younger infertile women may be related to the fact that ovarian reserve function changes less in women younger than 30 years old (20, 21). It has been reported that AMH level is considered to be an important indicator to predict ovarian responsiveness and is closely related to the occurrence of hyperresponsiveness and OHSS (22, 23). Our study also showed statistically significant differences in AMH values between groups with different ovarian responses. Kwee et al. (24) showed that AFC was another important indicator to predict ovarian responsiveness. When AFC >14, the sensitivity and specificity of ovarian hyperresponsiveness in IVF patients were 0.82 and 0.89, respectively. However, AFC is much less stable than AMH in different menstrual cycles of the same patient (25). Moreover, AFC can only be detected before the beginning of the cycle, although this does not hinder the choice of medication dosage.

Advocating individual ovulation stimulation is one of the principles of assisted reproductive technology. However, there is no unified standard for the prediction of ovarian responsiveness according to which indicators should be integrated and which models should be used. In the literature we reviewed, the prediction of ovarian responsiveness is based on the analysis of a single factor and the prediction of its specificity and sensitivity, such as age (26, 27) and AMH value (28). In this study, age, AFC, AMH, basal FSH, LH value on the trigger day of HCG, and diagnosis of EM and DOR were selected and analyzed as independent risk factors for different ovarian responsiveness to predict ovarian response earlier and achieve the best ovarian response during ovulation induction with the antagonist protocol. At the same time, we hoped to avoid low or high reactions, especially high reactions resulting in severe OHSS and other complications.

### Norman model predicts ovarian responsiveness

For patients entering the antagonist protocol, the baseline condition is fully evaluated, and each index corresponds to the score on the Norman model to obtain the baseline score. Subsequently, the LH value during ovulation induction and on trigger day were key factors for the total score. According to clinical experience and the Norman model, we can now find the appropriate LH value required on trigger day on the nomogram to obtain the appropriate probability of follicle number and avoid the risk of OHSS caused by obtaining too many follicles. Our model showed that those with an optimal ovarian response interval of 95% or less for the appropriate number of follicles were likely to have an excess number of follicles <30% of the time.

The Norman model can provide clinicians with an intuitive and quantitative predictive value of risk, which can be determined according to the comprehensive information of each patient. This makes this new reference protocol worthy of further testing; however, no nomogram is perfect. In the future, the predictive accuracy of the model may be improved by the addition of more indicators and data.

In conclusion, for different populations using the GnRH antagonist protocol to super-stimulate ovulation, we screened the factors that would allow the ovaries to achieve a normal range of response and avoid a high response, and successfully developed a Norman prediction model. We hope to be able to predict the outcome of ovulation hyperstimulation effectively, intuitively, and visually, which will provide value for clinical decision making. In addition to individualized ovulation induction therapy for patients of different ages and with different diagnoses, Gn dose can be adjusted in advance according to the specific conditions of patients. Similarly, the LH value on trigger day can be well controlled, the probability of obtaining the best number of oocytes can be predicted, and OHSS can be prevented in advance. According to this model, we can not only obtain the effectiveness and safety of treatment, but also maximize the time and economic benefits of patients, which is worthy of further improvement and promotion.

Of course, the study has some limitations. It was a retrospective analysis of only 3 years of cases. More studies for a longer time and more cases should be analyzed to create a more accurate Norman prediction model. In addition, all cases were done in the same reproductive center, which may ensure better control of the consistency of the procedure, but also compromise the generality of the results. Therefore, more cases and multicenter randomized controlled studies are needed to further evaluate the accuracy and feasibility of this model.

## Data availability statement

The original contributions presented in the study are included in the article/supplementary material. Further inquiries can be directed to the corresponding author.

## Ethics statement

The studies involving human participants were reviewed and approved by Committee of Fujian Provincial Maternal and Child Health Hospital. The patients/participants provided their written informed consent to participate in this study.

## Author contributions

Conception and design: WJ and XC. Administrative support: BZ. Provision of study materials or patients: SZ and XL. Collection and assembly of data: WJ and RL. Data analysis and interpretation: HZ and RL. Manuscript writing: WJ and HZ. Final approval of manuscript: All authors.

## Funding

This work was supported by the Fujian Provincial Natural Science Foundation Project of China (No.2021J05080).

## Acknowledgments

The authors thank the participation of all patients. The authors also thank the Charlesworth Group for language editing.

## Conflict of interest

The authors declare that the research was conducted in the absence of any commercial or financial relationships that could be construed as a potential conflict of interest.

## Publisher’s note

All claims expressed in this article are solely those of the authors and do not necessarily represent those of their affiliated organizations, or those of the publisher, the editors and the reviewers. Any product that may be evaluated in this article, or claim that may be made by its manufacturer, is not guaranteed or endorsed by the publisher.

## Abbreviations

AFC, Antral follicle count; AMH, Anti-Mullerian hormone; BMI, Body mass index; COH, Controlled ovarian hyperstimulation; DOR, Decreased ovarian reserve; HCG, Human chorionic gonadotropin; LH, Luteinizing hormone; OHSS, Ovarian hyperstimulation syndrome; PGT, Pre-implantation genetic testing.
